# Prospective data linkage to facilitate COVID-19 trials – A call to action

**DOI:** 10.23889/ijpds.v5i2.1383

**Published:** 2020-08-11

**Authors:** PA Paprica, MR Sydes, KM McGrail, AD Morris, MJ Schull, R Walker

**Affiliations:** 1 ICES, 2075 Bayview Ave, Toronto, ON, M4N 3M5, Canada; 2 Institute for Health Policy, Management and Evaluation, University of Toronto, 155 College Street, Suite 425, Toronto, ON, M5T 3M6, Canada; 3 Health Data Research Network Canada, 2206 East Mall, Vancouver, BC, V6T 1Z3, Canada; 4 MRC Clinical Trials Unit at UCL, Institute of Clinical Trials and Methodology, 90 High Holborn, University College London (UCL), London, WC1V 6LJ, UK; 5 Health Data Research UK, Gibbs Building, 215 Euston Road, London, NW1 2BE, UK; 6 Population Data BC, 2206 East Mall, Vancouver, BC, V6T 1Z3, Canada; 7 School of Population and Public Health, University of British Columbia, 2206 East Mall, Vancouver, BC, V6T 1Z3, Canada

The international research community is mobilising an unprecedented response to COVID-19, including more than 2500 registered clinical trials [1, 2]. There are also at least 100 observational studies based on administrative and routinely collected healthcare data [3, 4]. The small upfront effort to allow linkage of administrative data, registries, electronic medical records, and personal health records (collectively referred to as medical databases) to the trial data should create significant returns in scientific insight. However, to our knowledge, only a small number of COVID-19 trials are leveraging medical databases by linking to them.

The solution is simple and is a golden opportunity which should not be missed. As demonstrated by the West of Scotland Coronary Prevention Study (WOSCOPS) 20-year Follow-up Study [5], a major advantage of linkage is that administrative data can be used for follow up so that researchers can identify events that occur, including those that happen long after the study’s original time period for active assessment of participants has ended. More specifically, the WOSCOPS 20-year Follow-up Study found that statin treatment for five years in the original 1989 trial was associated with a legacy benefit, with improved survival and a substantial reduction in cardiovascular disease outcomes over a 20-year period in a high-risk cohort of men [6]. Further, the cost of follow-up assessment using medical databases was very low compared to the bespoke data collection of the original trial, costing on the order of tens of thousands rather than millions of dollars [7].

More recently, the UK RECOVERY trial of therapies for COVID-19 was the first to identify a survival benefit of dexamethasone in high-risk hospitalised patients with COVID-19. The study included linkage of trial-specific data and data from routine healthcare and registry sources. Using expedited access to existing medical databases, RECOVERY investigators were able rapidly to determine information on vital status (e.g., date and cause of death); discharge from hospital; intensive care use; and renal replacement therapy [8]. The RECOVERY trial’s speed and comprehensiveness deserve praise [9], both of which were enabled by linkage to medical databases.

Linkage to medical databases in trials can reduce cost (and thereby allow larger trials within an existing budget), increase speed (particularly if near real-time data flows can be achieved), support more complete long-term follow-up, and allow multiple outcomes to be monitored. In the case of COVID-19, it could also help researchers go beyond prospective analysis and forecasts based on COVID-19 cases, bringing in additional information about patient characteristics and past health system use. Combining clinical and health services research expertise will increase our understanding of the relevance of comorbidities, demographic factors and prior health system utilisation on the effects of COVID-19. As per the WOSCOPS 20-year Follow up Study example, linkage could also enable the assessment of the long-term impacts of interventions for COVID19, including experimental drugs and vaccines, years after clinical trials end, and at greatly reduced cost relative to bespoke data collection. Rare outcomes could be studied at scale and provide robust evidence regarding the safety of new vaccines, and thereby reassure regulators and the public that potential risks are carefully monitored [10].

There are, of course, caveats. Use of medical databases in trials requires full understanding of data quality limitations, excellent knowledge of database holdings, and case validation work [7]. It is also important that the public and trial participants support use of their data in trials [7, 11, 12]. Effective partnerships between trialists and medical database stewards will be essential, and Health Data Research UK (HDR UK) and Health Data Research Network Canada (HDRN Canada) have already begun to support this. For example, HDRN Canada organisations have developed standardised text that trialists can use when seeking participant informed consent for data linkage [13, 14], and the HDR UK Health Data Hub for clinical trials, NHS DigiTrials, delivered in partnership with NHS Digital, is providing services to improve the assessment of clinical trial feasibility, which has successfully contributed to the RECOVERY trial [15, 16].

Many countries around the world are creating COVID-19 trial datasets and databases of COVID-19-related data such as test results that cover entire populations. It is our strong recommendation, and sincere hope, that these data assets will be brought together through record linkage so that their scientific value and impact for society can be amplified. We call on trialists, data stewards and research funders to work together so that prospective linkage of trial data to medical databases becomes the norm, starting with COVID-19 trials. 
